# Global catalogue of microorganisms (gcm): a comprehensive database and information retrieval, analysis, and visualization system for microbial resources

**DOI:** 10.1186/1471-2164-14-933

**Published:** 2013-12-30

**Authors:** Linhuan Wu, Qinglan Sun, Hideaki Sugawara, Song Yang, Yuguang Zhou, Kevin McCluskey, Alexander Vasilenko, Ken-Ichiro Suzuki, Moriya Ohkuma, Yeonhee Lee, Vincent Robert, Supawadee Ingsriswang, François Guissart, Desmeth Philippe, Juncai Ma

**Affiliations:** 1 Institute of Microbiology, Chinese Academy of Sciences, Beijing, China; 2 World Data Centre for Microorganisms(WDCM), Beijing, China; 3 Belgian Coordinated Collections of Micro-organisms Programme, Belgian Science Policy Office, avenue Louise, 231 1050 Brussels, Belgium; 4 National Institute of Genetics, Yata, Mishima 411-8540 Japan; 5 Fungal Genetics Stock Center, University of Missouri, Kansas City, Missouri, USA; 6 G.K. Skryabin Institute of Biochemistry and Physiology of Microorganisms RAS, Pushchino, Moscow region, Russia; 7 Culture Collection Division Biological Resource Center (NBRC), National Institute of Technology and Evaluation (NITE), 2-5-8 Kazusakamatari, Kisarazu-shi, Chiba 292-0818 Japan; 8 Japan Collection of Microorganisms/ Microbe Divion, RIKEN BioResource Center, Koyadai 3-1-1, Tsukuba, Ibaraki 305-0074, Japan; 9 Seoul Women’s Unviersity/Korea National Research Resource Center, Seoul, Korea; 10 CBS-KNAW, Fungal Biodiversity Centre, Uppsalalaan 8, Utrecht, The Netherlands; 11 Bioresources Technology Unit, National Center for Genetic Engineering and Biotechnology, 113 Thailand Science Park, KlongLuang, Prathumthani 12120 Thailand

**Keywords:** Microbial resources, Data management, Data sharing

## Abstract

**Background:**

Throughout the long history of industrial and academic research, many microbes have been isolated, characterized and preserved (whenever possible) in culture collections. With the steady accumulation in observational data of biodiversity as well as microbial sequencing data, bio-resource centers have to function as data and information repositories to serve academia, industry, and regulators on behalf of and for the general public. Hence, the World Data Centre for Microorganisms (WDCM) started to take its responsibility for constructing an effective information environment that would promote and sustain microbial research data activities, and bridge the gaps currently present within and outside the microbiology communities.

**Description:**

Strain catalogue information was collected from collections by online submission. We developed tools for automatic extraction of strain numbers and species names from various sources, including Genbank, Pubmed, and SwissProt. These new tools connect strain catalogue information with the corresponding nucleotide and protein sequences, as well as to genome sequence and references citing a particular strain. All information has been processed and compiled in order to create a comprehensive database of microbial resources, and was named Global Catalogue of Microorganisms (GCM). The current version of GCM contains information of over 273,933 strains, which includes 43,436bacterial, fungal and archaea species from 52 collections in 25 countries and regions.

A number of online analysis and statistical tools have been integrated, together with advanced search functions, which should greatly facilitate the exploration of the content of GCM.

**Conclusion:**

A comprehensive dynamic database of microbial resources has been created, which unveils the resources preserved in culture collections especially for those whose informatics infrastructures are still under development, which should foster cumulative research, facilitating the activities of microbiologists world-wide, who work in both public and industrial research centres. This database is available from http://gcm.wfcc.info.

## Background

Microbial culture collections play an important and essential role in collecting, maintaining, and distributing quality assured living microbial strains. The Word Federation for Culture Collections (WFCC) is a Multidisciplinary Commission of the International Union of Biological Sciences (IUBS) and a Federation within the International Union of Microbiological Societies (IUMS).The WFCC promotes the interests of culture collections, develops shared resources, and organizes the International Conference on Culture Collections every three years. As one of its longstanding activities, the WFCC participated in the development of the WFCC World Data Centre for Microorganisms (WDCM) in the late 1960s [1]. With additional input from the United Nations Educational, Scientific and Cultural Organization Microbial Resources Centers (MIRCEN) project, the WDCM was maintained as the WFCC-MIRCEN WDCM and become accessible as an internet page in 1997. The WDCM serves as the data center of the WFCC and provides an important information resource for all microbiological activities. Additionally, the WDCM acts as a coordination center for data activities among WFCC members. As one of the main databases in WDCM, CCINFO (Culture Collection INFOrmation database) lists 652 culture collections from 70 countries maintain more than 1.9 million strains. (http://www.wfcc.info/ccinfo/, accessed 12/3/2013).

Increasing demands on culture collections for authenticated, reliable biological material and associated information were accompanied by the growth of biotechnology and basic science. The WFCC guidelines recommend that every collection publish an online or printed catalogue regularly, both to disseminate information about strains and to promote scientific and industrial usage of materials held in their collection. However, according to the available statistics, fewer than one-sixth of collections registered in CCINFO post their catalogue online and this greatly hinders the visibility and hence the accessibility of strains in these collections without public electronic catalogs.

To help all collections establish an online catalog, the WDCM has constructed a data management system and a global catalogue to organize, make public, and explore the data resources of its member collections. This data management system, called the WFCC Global Catalogue of Microorganisms (GCM) is a scalable, reliable, dynamic and user-friendly system that helps culture collections manage, disseminate and share the information related to their holdings. It also provides a uniform interface for the scientific and industrial communities to access the comprehensive microbial resource information.

## Construction and content

### Data sources

The Global Catalog of Microorganisms database contains information from a variety of sources:

• Information provided by culture collection staff

• Data from public data sources such as the US National Library of Medicine (PubMed) and the Patent database

• Links to external databases

• Tools for bioinformatics analysis including a search engine to enhance exploration of GCM data.

By the end of August 2013, the GCM contains strain information from 52 collections (Table 1) located in 25 different countries and regions. While the project is still in its construction phase, preliminary statistics describing the participating collections are unique and informative (Table 2).

**Table 1:** Participant list of GCM collections

**Acronym**	**Full name**	**Country**
BCC	BIOTEC Culture Collection	Thailand
BCCM/DCG	BCCM Diatom Collection Gent	Belgium
BCCM/IHEM	Belgian Coordinated Collections of Microorganisms / IHEM Fungi colleciton	Belgium
BCCM/LMBP	Belgian Coordinated Collections of Microorganisms / LMBP Plasmid Collection	Belgium
BCCM/LMG	Belgian Coordinated Collections of Microorganisms/ LMG Bacteria Collection	Belgium
BCCM/MUCL	Mycotheque de l’Universite catholique de Louvain	Belgium
BCCM/ULC	BCCM/ULC Culture Collection of (sub)polar cyanobacteria	Belgium
BCRC	Bioresource Collection and Research Center	Chinese Taipei
BIM	Belarusian Collection of non-pathogenic microorganisms	Belarus
CBS	Centraalbureau voor Schimmelcultures, Filamentous fungi and Yeast Collection	Netherlands
CCAP	Culture Collection of Algae and Protozoa	U.K.
CCARM	Culture Collection of Antimirobial Resistant Microorganisms	Korea
CCCryo	Culture Collection of Cryophilic Algae	Germany
CECT	Coleccion Espanola de Cultivos Tipo	Spain
CGMCC	China General Microbiological Culture Collectio Center	China
CIP	The Collection of the Institut Pasteur	France
CIRM-CF	Centre International de Ressources Microbiennes - Champignons Filamenteux	France
CIRM-CFBP	Centre International de Ressources Microbiennes - Levures (CLBP)	France
CIRM-Levures	Centre International de Ressources Microbiennes - Levures	France
CM-CNRG	Coleccion de Microorganismos del Centro Nacional de Recursos Geneticos	Mexico
CVCM	Centro Venezolano de Colecciones de Microorganismos	Venezuela
CWU-MACC	Herbarium of Kharkov University (CWU) – Micro Algae Cultures Collection	Ukraine
DMic	Medical importance fungi culture collection	Argentina
DSMZ	Leibniz-Institut DSMZ-Deutsche Sammlung von Mikroorganismen und Zellkulturen GmbH	Germany
FACHB	Freshwater Algae Culture Collection, Chinese Academy of Sciences	China
FGSC	Fungal Genetics Stock Center	USA
Fiocruz-CLIOC	Coleção de Leishmania do Instituto Oswaldo Cruz	Brazil
GDMCC	Guangdong Culture Collection Centre of Microbiology	China
HPKTCC	Helicobacter pylori Korean Type Culture Collection	Korea
IMI(CABI)	CABI Genetic Resource Collection	U.K.
ITDI	Industrial Technology Development Institute Microbial Culture Collection	Philippines
ITM	Belgian Coordinated Collections of Microorganisms Mycobacterial Culture Collection	Belgium
JCM	Japan Collection of Microorganisms	Japan
KCTC	KCTC Korean Collection for Type Cultures	Korea
KEMB	Korea national Environmental Microorganisms Bank	Korea
KMMCC	Korea Marine Microalgae Culture Center	Korea
LEF	Korea Lichen & Allied Bioresource Center	Korea
LIPIMC	Lembaga Ilmu Pengetahuan Indonesia , Indonesian Institute for Sciences	Indonesia
MCC-MNH	Microbial Culture Collection - Museum of Natural History, Museum of Natural History (MNH)	Philippines
NBRC	NITE Biological Resource Center	Japan
PNCM	Philippine National Collection of Microorganisms	Philippines
PVGB	Plant Virus GenBank	Korea
TISTR	TISTR Culture Collection, Bangkok MIRCEN	Thailand
UCCAA	Ukrainian Collection of Cholera Aetiological Agents O1 and non O1 serogroups	Ukraine
UCDFST	Phaff Yeast Culture Collection	USA
UL	The UNILAB Clinical Culture Collection, United Laboratories	Philippines
UMinho-MUM	Micoteca da Universidade do Minho	Portugal
UOA/HCPF	UOA/HCPF University of Athens/Hellenic Collection of Pathogenic Fungi	Greece
UPCC	Natural Sciences Research Institute Culture Collection	Philippines
UPMC	MICROBIAL CULTURE COLLECTION UNIT	MALAYSIA
VKM	All-Russian Collection of Microorganisms	Russia
VTCC	Vietnam Type Culture Collection	Vietnam

**Table 2:** Summary of GCM strain data

**Organism type**	**Species number**	**Strain number**	**Type strain**	**Sequences**	**Publications**	**Patents**
Antibody	7	33	0	0	0	0
Phage	181	239	0	0	1	0
Virus	33	296	0	0	0	0
Cyanobacteria	134	287	0	178	0	0
Protozoa	236	754	0	0	0	0
Actinomycetes	842	1490	0	271	192	9
Archaea	1410	3273	1165	2176	1573	48
Microalgae	1820	5495	4	2	1	1
Plasmid	2030	2030	0	0	5	9
Yeast	3668	34907	4796	54773	2089	98
Bacteria	13714	101395	14233	29304	10975	268
Fungi	18537	121548	29916	94348	1960	65
Diatom	19	242	0	0	0	0
Mycobacteria	50	214	0	0	0	0
Other**/**Rotifera	755	1730	0	0	0	0
Total	43436	273933	50114	181052	16796	498

Information was as submitted by individual collections. Sequence, publication, and patent data were extracted from Genbank, Pubmed, Patent database using the strain numbers.

The GCM implements the WDCM Minimum Data Sets (MDS) and Recommended Data Sets (RDS) based on widely applied standards such as the OECD Best Practice Guidelines for Biological Resource Centres [2], the Microbial Information Network Europe (MINE) [3], as well as the Common Access to Biological Resources and Information (CABRI) [4]. A detailed description, together with examples of 15 WDCM MDS items can be found at http://gcm.wfcc.info/datastandards/index.jsp (last accessed 12/3/2013).To build the GCM, each participating collection transferred their catalogue information by one of several pathways. Some collections sent Excel or XML files while others provided direct access to their database files. WDCM integrated the data into a global dataset, processed the data to identify relationships among collections (for example strains held in multiple collections), and published the strain information on the GCM web page (http://gcm.wfcc.info). Because not all collections use the same data schema, some of the data items provided by culture collection staff were manually reclassified by GCM staff to allow for an easier integration of catalogue information.

Publications concerning strains are collected from PubMed using both strain number and species name for keyword queries. Nucleotide sequences are extracted from GenBank [5], protein sequence data are collected from UniProt [6], and information about protein 3D structure are extracted from the PDB database [7]. Genome sequencing information is collected from NCBI Microbial Genomes Resources (NCBI).

### Organization of data

**Figure 1:**
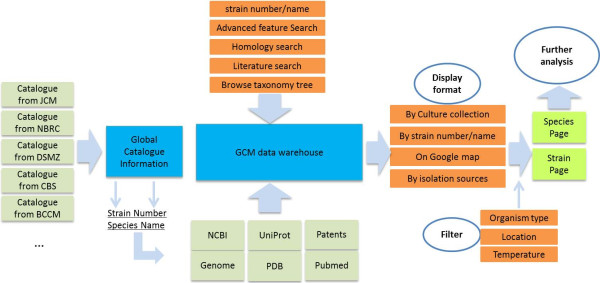
**Scheme of the workflow of GCM.** Catalogue information from each of collection, shown on the left, is used to construct the framework of the global catalogue database. Species name and strain numbers are collected from the catalogue information, and are further used to identify and extract information from public database such as Genbank, NCBI Genome, SwissProt, PDB, Pubmed and the patent database. The data warehouse is built in a SQL database, and can be accessed via a web interface through different search options. Search results may be displayed in different formats allowing users to refine the results by using filters. The final results are displayed either as a strain page or they can be gathered into a species page depending on the query. BLAST and ClustalW are provided for further analysis of the results.

The GCM database contains the following fields for each strain entry: strain number, other collection numbers, name, organism type, history of deposition, date of isolation, isolation sources, geographic origin, status, optimal temperature for growth, minimum temperature for growth, maximum temperature for growth, medium, application, and published citations to the use of the strain. In addition to these WFCC MDS entries, the GCM contains extensive citation, patent, and gene or genome information related to each strain. All of this information is available from the strain information page for each strain. A schema of the data flow of GCM is shown in Figure 1.

**Figure 2:**
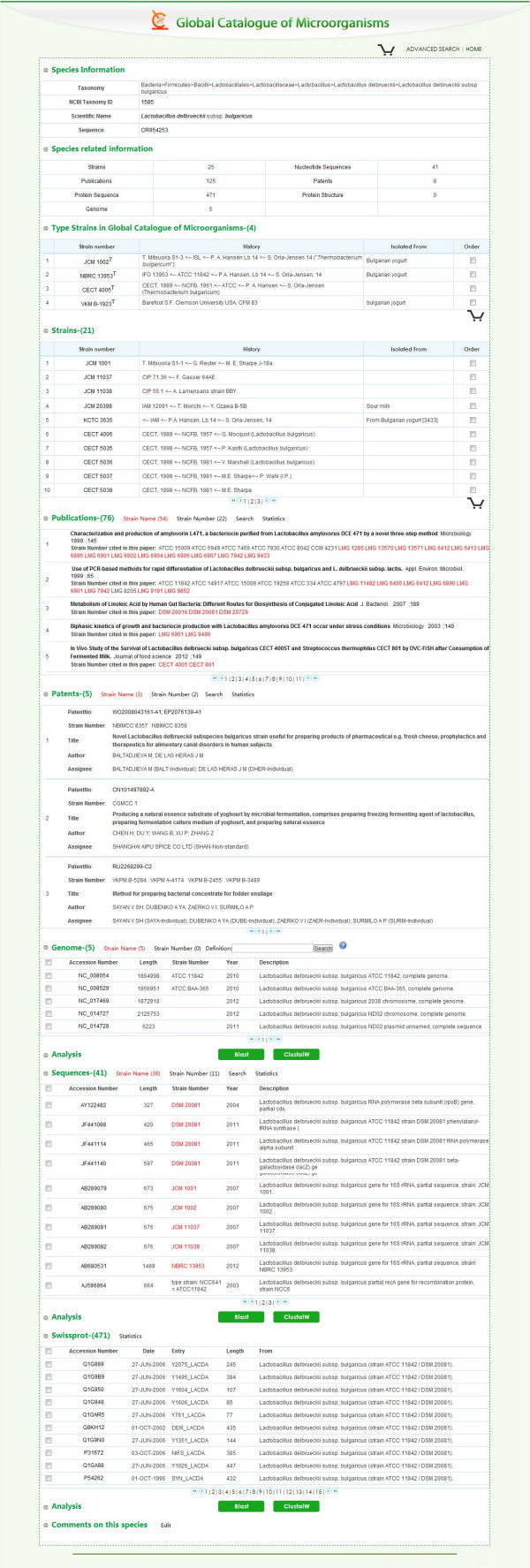
**Example of Species page of *Lactobacillus delbrueckii *** **subsp. *bulgaricus.***

Strains belonging to the same species as well as sub-species are automatically associated to form a species page (Figure 2). A taxonomic tree of species 2000 [8] is generated to serve as a reference for taxonomic identification. Type strains, indicated by their collections are listed on species page. Data on individual strains are organized by culture collections location, type of strain, isolation sources, and genus and species as well. As a result, all data can be retrieved through the browse option provided in the web server according to these properties.

Metagenome and Microbes Environmental Ontology (Hiroshi Mori [9]) which is an ontology about microbial environment was used for text mining of values of isolation sources. The text contained in this data item was automatically compared with the terms of MEO and then sorted into 13 different categories such as soil, microbial-mat/Biofilm, or host-associated, among others (Table 3). For the values that could not be automatically assigned to a specific category, manual curation is required. Data concerning environmental habitats of the isolates can provide important information about the diversity of organism types that are related with certain isolation source types.

**Table 3:** Isolation sources of Strains sorted by type of organism

**Isolation source type**	**Fungi**	**Bacteria**	**Yeasts**	**Actinomycetes**	**Archaea**	**Phage**	**Microalgae**	**Total**
Sludge/Wasterwater	1	1091	6	-	9	-	2	1109
Soil	1708	3468	484	264	95	1	1	6021
Sediment	4	46	17	-	14	-	-	81
Fermentation products	123	358	327	-	1	-	-	809
Plant-associated	405	314	644	-	1	-	2	1366
Host-associated	139	480	180	-	3	-	-	802
Human-associated	18	11167	55	-	15	-	2	11257
Water	4	398	50	-	48	-	-	500
Microbial-mat/Biofilm	-	-	-	-	1	-	-	1
Air	6	20	29	-	1	-	-	56
Genetic engineering strain	22698	-	-	-	-	-	-	-
Food	193	83	69	-	2	-	-	347
Others	135	728	76	-	25	-	1	965
Total	2736	18153	1937	264	215	1	8	23314

About 48% of the strains have geographic information and these strains are from 164 different countries or regions. Data on the geographic origin of isolates (Table 4) is complementary to the habitat and can provide useful information on relative biodiversity and sampling efforts for different countries and regions. These data will ultimately be integrated into the Global Biodiversity Information Facility (GBIF) database through planned activities of GCM (Éamonn [10]).

**Table 4:** Top 20 countries from which strains were collected

**Order**	**Country**	**Counts**	**Order**	**Country**	**Counts**
1	Japan	8248	11	China	3429
2	France	8070	12	India	2907
3	United States	7701	13	Russian Federation	2872
4	Netherlands	6709	14	South Africa	2419
5	Korea	6270	15	Italy	2009
6	Germany	6051	16	Canada	1848
7	Thailand	5894	17	VietNam	1818
8	United Kingdom	5717	18	Sweden	1786
9	Belgium	5177	19	Australia	1695
10	Spain	3869	20	Switzerland	1466
Total		85955			

114,578 of 273,933 strains contain information regarding their geographic origins. The strains were collected from 164 countries and regions, of which, 85,955 strains were collected from only 20 countries. This takes up approximately 74% of total strains, which indicates a relatively high sampling effort in these countries.

### Data quality control

Because original catalogue data are sometimes non-validated, quality control measures are necessary before data can be published in GCM online. The most frequent quality problem is the misspelling of species name or non-standard naming of species. For example, “*Absidiapsychrophilia*” was wrongly spelled as “*Absidiapsychrophila*” in certain collections. In such cases, GCM uses standard microbial nomenclature databases to perform a quality check of its taxonomic data. Databases include the List of Prokaryotic names with Standing in Nomenclature (LPSN) [11], “Species 2000”, and NCBI taxonomy [12] for bacteria and archaea, MycoBank [13] for fungi and yeast.

A programming script was written (in the Java™ language) to automatically compare species names between the GCM catalogue and the nomenclature databases cited above. The comparison showed that from the 36,340 different archaea, bacteria, fungi and microalgae contained in GCM, 2188 could not be found in any of the nomenclature databases above. The average mismatching is 6% (Table 5). When conflicts are identified, GCM sends the results of these comparisons to curators at the relevant collections to allow them to edit their catalogue information online. When mismatches occur, the system provides the probably correct species name based on character string similarity. Following such comparison, the majority of spelling mistake is corrected.

**Table 5:** Result summary of species name check

**Organism type**	**Species names**	**Un-matched species name**	**Percentage of un-match**
Archaea	1399	32	2.30%
Microalgae	1457	360	24.70%
Fungi	20719	698	3.40%
Bacteria	12855	1098	8.50%
Total	36430	2188	6.00%

This table provides comparative results of the species names within GCM with public microbial nomenclature database. Species2000, NCBI taxonomy, LPSN and Mycobank were used as reference databases. The average percentage of unmatched names is 6%, while the archaea and fungi showed lower than average percentage of unmatched names. The percentage of unmatched names is relatively high for microalgae, possibly due to the irregular naming for microalgae.

The second type of problems with the quality of information is related to data content. For example, some “*Escherichia coli*” strains were wrongly assigned as “Fungi” in the host collection databases. The GCM system collects and compares the lists of differences in the description of cultures in one collection with cultures of the same strains in other collections.

History information was used to do the quality check for species name as well. Totally 12147 strains contain detailed history information in GCM. The system listed all of species name and compared with their history species name in other collections. The result indicated that among 12147 strains, 1746 strains had different species name with their history strains. Further analysis on the result showed that, among the mismatch, 267 belonged to misspelling problems such as “*Candida viswannathii*” was wrongly spelled to “*Candida viswanathii*”. However, the left were mistakes or name changes occur during the strain transfer between collections.

Divergent results are forwarded to the curators of the respective collections for corrections. Performing such controls for all fields of the database greatly assist collections in correcting existing mistakes.

## Utility

### Interface and web tools

The database homepage contains a world map which indicates the countries and regions that have already joined the GCM project. Statistics and graphics indicate the continuing acquisition of data into the GCM. A simplified search interface allows the querying of the database by using the strain number and species name. In addition, a variety of tools have been implemented to enhance its use. The main web tools that were integrated into the GCM are the following:

### Advanced search

Three query options are available in the advanced search section. Users may search strains within a range of values for one or several properties, including cultivation temperature, substrate, or application, before retrieving the retrieve corresponding results.

Since GCM maintains nucleotide sequences data associated with individual strains, a sequence alignment tool based on the Basic Local Alignment Search Tool (BLASTN) [14] is included. Results are ordered by similarity.

Bibliographic and patents queries are also possible and allow users to search by keywords in titles, abstracts of articles or patents. Search results are listed as strain numbers, strain names, publication abstracts and titles and can be exported in text file format.

With the advanced search tools, the system can perform the following searches

➢ Searching for type strains for some taxa in certain culture collections

➢ Searching for strains with specific characteristics in the list of Culture Collection (CC) or Biological Resource Center (BRC), such as range of growth temperature, transfer history, collected location and others

➢ Searching for strains with specific properties

➢ Searching strains isolated from various substrates, including sludge or wastewater, soils, sediment, fermentation products. Results are listed in table format, with the type of organism type used as column name;

➢ Searching strains with particular protein coding genes

Results are listed by strain number, species name, culture collections, and isolation sources. A few filter windows are provided in the result page to allow users to refine the results by collections, growth temperature, isolation sources or organism type.

### Species tree viewer

A species2000 taxonomy tree is used for the organization of strain information. Species names are used to map between GCM data and species2000 name (http://www.sp2000.org/), and then a taxonomic tree containing the number of strains for each genus is constructed. User can then browse the taxonomy tree itself, or search a species name within it.

### Map viewer

**Figure 3:**
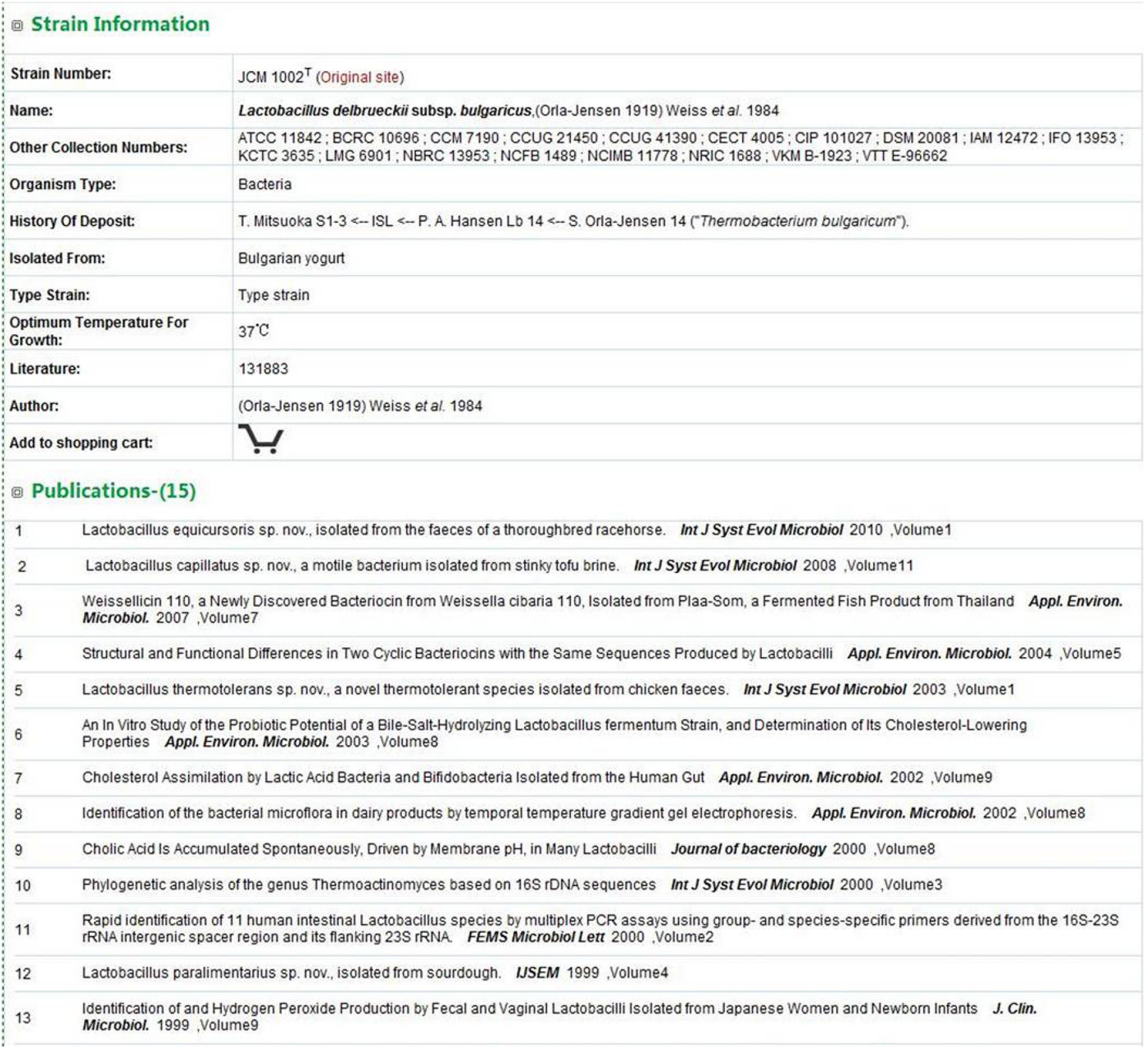
**Example of strain information of *Lactobacillus delbrueckii *** **subsp. *bulgaricus.***

While geographic origins of strains are usually provided as rural location, national park or cities, GCM can automatically translate such locations into more precise information of longitude and latitude. Strains are then displayed on a map using the Google maps API. In some cases, the location information is a more specific place such as a university or an institute, which could not be translated directly into longitude and latitude values. In such cases, manual annotation by the administrator of GCM will then use the value of the located city as an approximation. An example strain information page is displayed in Figure 3.

### Data analysis

A variety analysis tools are also employed on both the strain information and species page. The BLAST program (Altschul SF [13]) was used for sequence homology searches within the database. For sequences related to the same strain or species, the ClustalW [15] program is provided to perform multiple sequence alignment analysis.

### Data update and management

**Figure 4:**
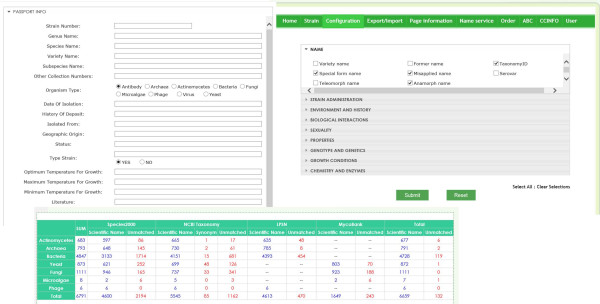
**Database management system for collections.** Users can import data by generating an EXCEL file to meet the WDCM RDS. After the data are imported into the system, users can either update or edit the catalogue information online. A species name check result is provided in the database management system to provide an overview of data quality and allow for further modifications.

To provide the greatest benefit to partner collections, a database management function was provided to GCM participating collections (Figure 4). After registration with the GCM project and filling out a metadata form, a user account will be given to the collection. Curators can then either export catalogue information in batch or add strain information individually. The system automatically records every operation, including updates, additions or deletions and after approval by the administrators in charge, the updated records are published online.

## Discussion and conclusion

A large amount of microbial resources are preserved as living strains in collections, however, information describing these strains is often unavailable. Each culture collection is independently responsible for the maintenance of data associated with their microbes, there is presently no enforced data harmonization and information sharing mechanism is available. Such situation hinders both the efficient management of collections and the ability to explore statistics about world microbial resources. Therefore, there is great demand for developing a mechanism for digital, online resource sharing, which provides a fundamental tool for best practices in information management.

The major target group for such system are culture collections staff, as well as academic and industrial microbiologists. We believe that GCM will assist collections, which lack the required human resources and information technology, to publish their stock information in an efficient and standardized way that is most useful for scientific and industrial communities. Database queries via a user-friendly and web-based interface should greatly promote the sharing and use of microbial resources.

While this project is still in its early stage, we are confident that it will continue to grow with the further addition of data, analytical tools and other functionalities. In the future, additional database management tools will be provided to allow more culture collections to share their data via GCM. These tools will lead to the increased availability of accessible data pertaining to microbial strains held in public collections and their utilization for bioindustry, medicine, and research. As it grows, GCM will incorporate information related to enzymatic and metabolic pathways using developing genomics and bioinformatics tools. Ultimately, GCM is a comprehensive data platform on microbial resources that is available to the public.

## Availability and requirements

The GCM database runs on a platform with both Java and MySQL server. Catalogue information gathered from associated collections is centralized within WDCM servers, which is hosted at the Institute of Microbiology, of the Chinese Academy of Sciences.

The Blast program is used for the sequence homology search in the database (BLASTN 2.2.25). Multiple sequence alignments are performed using the ClustalW program (version2.1). GCM is available at http://gcm.wfcc.info.

## Competing interest

The authors declare that they have no competing interest.

## Authors’ contributions

LW designed database and web services. QS integrated data resources from Pubmed and Genbank. SY developed the database, made the webpages and wrote the database queries. HS designed and supervised construction of the database. YZ, KM, AV, SKI, MO, YL, VR, SI and FG provided the catalogue information and participated in the design of the database. LW and KM wrote the manuscript. DP and JM designed the database and supervised construction of the project. All authors read and approved the final manuscript.
